# Tissue-specific silencing of integrated transgenes achieved through endogenous RNA interference in *Caenorhabditis elegans*

**DOI:** 10.1080/15476286.2024.2332856

**Published:** 2024-03-26

**Authors:** Siyu Chen, Weihong Liu, Lei Xiong, Zhiju Tao, Di Zhao

**Affiliations:** a Tianjin Key Laboratory of Exercise Physiology and Sports Medicine, Institute of Sport, Exercise & Health, Tianjin University of Sport, Tianjin, China; b School of Life Sciences, Tsinghua University, Beijing, China; c Intelligent Perception Lab, Hanwang Technology Co. Ltd, Beijing, China; d Computer Science and Artificial Intelligence Lab, Massachusetts Institute of Technology, Cambridge, MA, USA; e The Broad Institute of Harvard and MIT, Cambridge, MA, USA; f MOE Key Laboratory of Bioinformatics, Beijing Advanced Innovation Center for Structural Biology, Center for Synthetic and Systems Biology, Tsinghua-Peking Center for Life Sciences, School of Life Sciences, Tsinghua University, Beijing, China

**Keywords:** *C. elegans*, transgene silencing, endo-RNAi, tissue-specific

## Abstract

Transgene silencing is a common phenomenon observed in *Caenorhabditis elegans*, particularly in the germline, but the precise mechanisms underlying this process remain elusive. Through an analysis of the transcription factors profile of *C. elegans*, we discovered that the expression of several transgenic reporter lines exhibited tissue-specific silencing, specifically in the intestine of *C. elegans*. Notably, this silencing could be reversed in mutants defective in endogenous RNA interference (RNAi). Further investigation using knock-in strains revealed that these intestine-silent genes were indeed expressed *in vivo*, indicating that the organism itself regulates the intestine-specific silencing. This tissue-specific silencing appears to be mediated through the endo-RNAi pathway, with the main factors of this pathway, *mut-2* and *mut-16*, are significantly enriched in the intestine. Additionally, histone modification factors, such as *met-2*, are involved in this silencing mechanism. Given the crucial role of the intestine in reproduction alongside the germline, the transgene silencing observed in the intestine reflects the self-protective mechanisms employed by the organisms. In summary, our study proposed that compared to other tissues, the transgenic silencing of intestine is specifically regulated by the endo-RNAi pathway.

## 1. Introduction

Most transgenes in animals and plants encounter rejection by the host, which is an important protective strategy for organisms [1–8]. When multiple copies of a transgene are present, stable transgenerational silencing in both germline and somatic cells can be triggered [9,10]. In the case of *C. elegans*, a specific type of transgenic silencing known as co-suppression occurs, where the presence of multiple-copies of a transgene leads to the silencing of the corresponding endogenous gene in the germline, relying on the included coding sequence [11,12]. This phenomenon of transgene silencing intersects with the molecular machinery of RNAi [10,11]. RNAi is not only a highly conserved mechanism for gene silencing in response to double-stranded RNA (dsRNA) [13,14], but it also plays a role in regulating endogenous gene expression through endo-small interfering RNAs (siRNAs).

*C. elegans* possesses a diverse array of small regulatory RNAs, typically consisting of 20–30 nucleotides. These small regulatory RNAs play crucial roles in various biological processes by modulating the expression of target genes [15,16]. Specifically in *C. elegans*, endo-siRNAs, and other small RNAs collectively contribute to genome defence by targeting and suppressing deleterious or foreign transcripts, including transposons and transgenes [17–19]. In contrast to exo-RNAi, which is triggered by exogenous dsRNA and results in gene silencing [20], endo-RNAi is initiated by endo-siRNAs. Endo-siRNAs were first identified in *C. elegans* but have since been uncovered in a wide range of eukaryotic organisms [21–25]. In *C. elegans*, these endo-siRNAs exhibit complementarity to both coding and noncoding sequences and are found throughout significant portions of the genome [22]. Extensive studies have demonstrated that the biogenesis of endo-siRNAs relies on multiple proteins, including the RRF-3 (RNA-dependent RNA polymerase-3), EGO-1 (Enhancer of Glp-One), and RRF-1 (RNA-dependent RNA polymerase-1) [18,26], as well as the exonuclease ERI-1 (exoribonuclease-1), and the dicer enzyme DCR-1 (DiCer Related-1) [27,28]. DCR-1, functioning as an RNase III enzyme, converts dsRNAs into small RNAs. It is believed that endo-siRNAs play a negative regulatory role in the expression of homologous mRNAs.

Deep sequencing of endogenous small RNAs has provided insight into the expanded repertoire of microRNAs in *C. elegans* [29]. Among the endo-siRNAs, there are 5’guanosine antisense small RNAs that consist of 26 and 22 nucleotides (nt) [22], referred to as 26 G RNAs, and 22 G RNAs respectively. The synthesis of 26 G RNAs primarily mediated by RRF-3 and subsequently processed by Dicer. These 26 G RNAs are notably enriched in the male and female germline [18]. In contrast, 22 G RNAs are generated through unprimed RNA synthesized by RRF-1 and EGO-1 [26,30]. These 22 G RNAs constitute another abundant class of endogenous small RNAs, with corresponding sequences antisense to more than 50% of annotated genes [26]. It is worth noting that the production of 22 G RNAs is robustly triggered by 26 G RNAs, although it is not the sole mechanism underlying the biogenesis of 22 G RNAs [31].

In *C. elegans*, fragments of germline genes are typically safeguarded through the targeted suppression by siRNA production, which is essential for proliferation [32]. Our study demonstrates that several transcription factors previously believed to be involved in housekeeping functions are actually specifically silenced in the intestine during the L1 stage *C. elegans*, as revealed by the comprehensive transcription factor expression atlas encompassing the entire organism [33]. Subsequent investigations have revealed that the silencing of these transcription factors in single-copy transgenes is not associated with exo-RNAi. Instead, we observed a de-silencing effect when essential components of the endo-RNAi pathway or histone modification factors were disrupted. Our findings indicate that the intestine-specific silenced state is mediated through the endo-RNAi pathway. Notably, *mut-2*, *mut-7*, and *mut-16*, three critical factors known to influence intestine de-silencing, exhibit high expression levels in the intestine. This suggests that the mechanisms underlying transgene silencing in different tissues are distinctly regulated.

To sum up, the current study is aimed at unveiling the mechanisms underlying the transgenic silencing of *C. elegans* intestine.

## 2. Materials and methods

### 2.1. Mutant strains

*C. elegans* strains were derived from Bristol N2 [34]. All strains used in this study were raised on Nematode Growth Medium (NGM) plates and fed OP50 *E. coli* at 20°C in incubators, unless otherwise noted. The following mutants were used in this work: *rde-1(ne219)*V, *rde-4(ne301)*III, *rde-11(hj37)*IV, *rde-10(hj20)*I, *sid-1(pk3321)*V, *mut-2(ne298)*I, *rde-12(ust17)*V, *mut-16(tm3748)*I, *rsd-2(tm1429)*IV, *nrde-3(tm1429)*X, *mut-7(pk204)*III, *ergo-1(gg98)*V, *nmad-1(ok3133)*III, *set-25(n5021)*III, *met-1(n4337)*I, *met-2(n4256)*III, and *rrf-1(pk1417)*I.

### 2.2. Reporter strain construction

Promoter sequences were defined as the intergenic sequences upstream the start codon using the criteria as previously described [35]. The single-copy transgenic reporter strains were generated using MosSCI as described previous [36]. Each promoter:H1:mCherry fragment was cloned into pCFJ350 plasmid, then it was injected into EG4322 worms along with pJL43.1 (Mostase coding vector). Two selection markers pCFJ90 (P*myo-2*::mCherry) and pCFJ104 (p*myo-3*::mCherry) were co-injected to help to screen integrated transgenic worms. The single-copy insertion transgenic strains used in this study are as follow:

*thuSi24[pisw-1:H1:mCherry]*, *thuSi44[plin-13:H1:mCherry]*, *thuSi45[plsy-27:H1:mCherry]*, *thuSi21[prnf-113:H1:mCherry]*, *thuSi20[pbed-1:H1:mCherry]*, *thuSi55[patf-2:H1:mCherry]*, *thuSi10[pF38C2.7:H1:mCherry]*, *thuSi128[pmut-2:H1:mCherry]*, *thuSi131[pmut-16:H1:mCherry] thuSi134[pmut-7:H1:mCherry]*.The multiple-copy transgenic strains were generated by microparticle bombardment using *unc-119* as a selection marker, as previously reported [37]. For knock-in strains of *isw-1* and *lin-13*, the reporter sequence was inserted into the C-terminus of the genomic locus of each transcription factor using the CRIPSR/Cas9 system as described previously [38], to generate: *thu81[isw-1:SL2:H1:mCherry]*, *thu82[lin-13:SL2:H1:mCherry]*. Endogenous transcription factor coding sequence and reporter sequence were separated by trans-splicing acceptor SL2 sequence [39]. Single guide RNA sequences used in this study are as follow: *isw-1*, 5’-atttagcagtaatcaaaggg-3’; *lin-13*, 5’-ttaatccagtgtcagaactt-3’.

### 2.3. Live worm imaging and florescent measurement

Worms hatched within 3 hours were collected as early stage L1 larvae. For live imaging, worms were placed onto 2% agarose pads on glass slides and immobilized using levamisole (2.5 mg/mL). Images were captured using a Zeiss Imager A2 epifluorescence microscope. The expression level of florescent proteins was calculated using the ZEN 2011(Zeiss, Jena, Germany).

### 2.4. Intestine-depletion score

We calculated the mean expression level for each housekeeping gene across eight central tissues, generating tissue expression values. From these values, we identified the smallest expression level and subtracted it from all other tissue values. This transformation allowed us to differentiate the most depleted genes across various tissues. Finally, we generated a heatmap to display the expression values observed in the intestine.

### 2.5. Statistical analysis

Data were presented as mean ± SD and analysed using GraphPad Prism software. All experiments were conducted in triplicate. Student’s t-test was applied to compare between two groups, and one-way analysis of variance (ANOVA) or two-way ANOVA was used to compare the differences of more than two groups. A *p*-values less than 0.05 was considered to be statistically significant.

## 3. Results

### 3.1. Intestine silencing in integrative transgenic reporter strains of *C. elegans*

To investigate the expression patterns of transcription factors in *C. elegans*, we generated reporter strains through bombardment [37], enabling the visualization of gene expression at the single-cell level [40]. These reporter strains utilized promoter sequences fused with fluorescent proteins, allowing for the detection of transcription activities. Subsequently, L1 worms from each reporter strain were analysed to determine their expression patterns in terms of tissues and cells.

**Figure 1.**
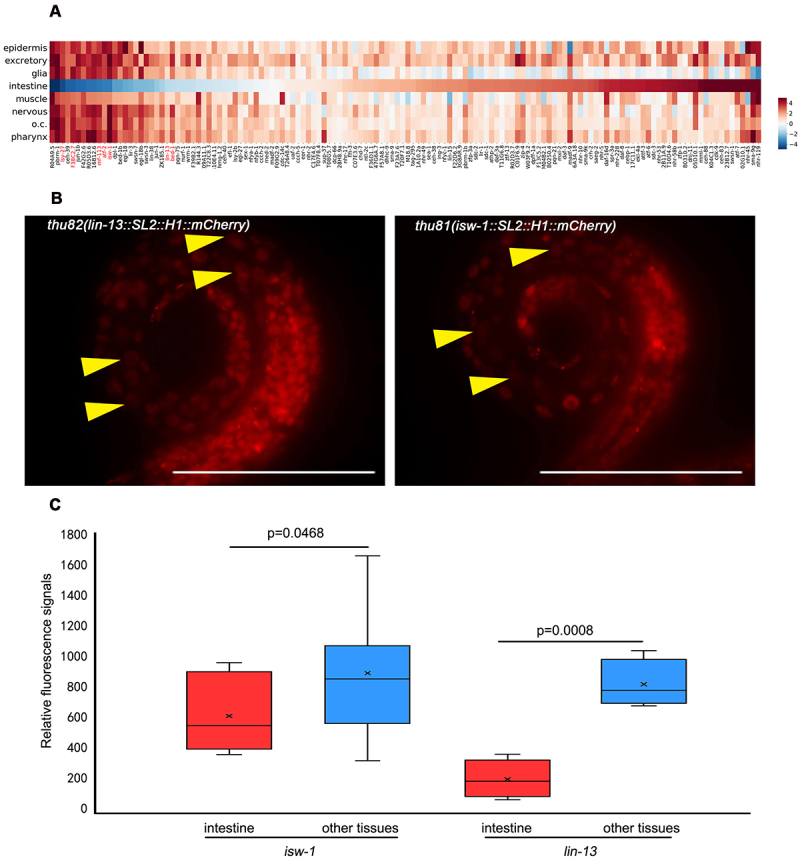
The varied expression patterns of housekeeping transcription factors in transgenic and endogenous knock-in lines. (a) The tissue-level expression pattern of housekeeping genes arranged by intestine depletion scores. Each column indicates a transgenic line generated by bombardment, and each line indicates a tissue. The red marked genes are also tested by MosSCI in further study. (b) Two typical housekeeping genes expressed in all tissues of worm including intestine, when the endogenous knock-in strain was examined. The reporter lines were generated by CRISPR/Cas9, and the fluorescent mCherry sequence was inserted into the 3’ end of those genes. The worms shown here are at the L1 stage. Data were collected over three independent experiments. Yellow arrowheads indicate intestine nuclei. Scale bar, 50 μm. (c) For the knock-in strains, quantification of fluorescence signal was performed between the intestine and other tissues. Student’s t-test was applied for statistical analysis.

All 558 cells of worms were manually annotated, and we defined genes expressed in at least 473 of 558 cells at the L1 stage worm as housekeeping genes. In this study, we report the intestine-specific silencing of the reporters among the housekeeping genes (Figure 1(a); Supplementary Data 1). Previous studies have shown that repetitive arrays in the germ line of *C. elegans* are consistently silenced [2,41]. To eliminate the potential influence of varied transgene copies, we generated single-copy intrachromosomal transgenes of seven housekeeping genes using MosSCI, employing the identical promoters as those used in the bombardment [42]. Furthermore, transgenes generated by MosSCI exhibit similar neighbour chromosome contexts. Most of the randomly selected single-copy transgenes among housekeeping genes remain silent (Table 1). These results suggest that the intestine-specific transgene silencing is not caused by multiple transgene copies.

**Table 1.** The intestine silencing in multi-copy and single-copy transgene reporter lines.

gene	Bombardment	MosSCI
*atf-2*	Silence	Silence
*bed-1*	Silence	Silence
*F38C2.7*	Silence	NA
*isw-1*	Silence	Silence
*lin-13*	Silence	Silence
*lsy-27*	Silence	Silence
*rnf-113*	Silence	Not silence

However, the endogenous mCherry knock-in strains of *lin-13* and *isw-1* exhibit noticeable fluorescent signals in the intestine (Figure 1(b); Figure S1). Throughout the development from L1 to adult, these two transgenes were expressed in the intestine at levels comparable to other tissues (data not shown). This observation suggests that transgenes of genes expected to be expressed *in vivo* may be subject to tissue-specific silencing.

### 3.2. Intestine de-silencing is significantly affected by endo-RNAi mutants, whereas exo-RNAi mutations maintain intestine silencing

We utilized *lin-13* and *isw-1* single-copy transgenic reporter lines to screen for mutants that could restore transgene repression in the intestine. Previous studies have indicated that chromatin modification factors and the RNAi pathway are potentially associated with intestine-specific silencing.

**Figure 2.**
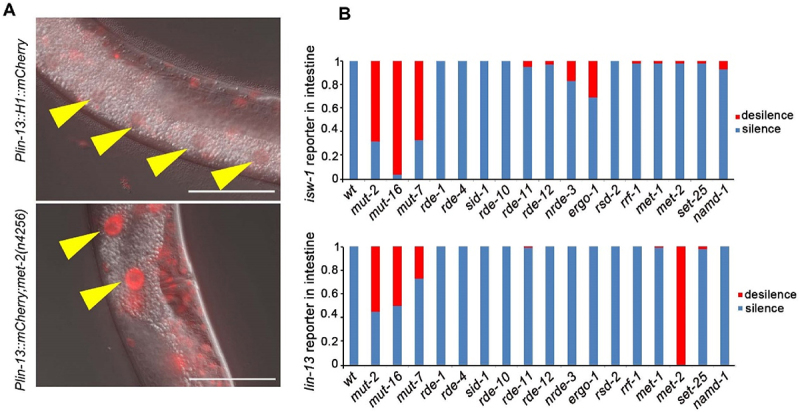
Intestinal silenced transgenes are de-silenced by mutators and *met-2*. (a) *lin-13* reporter line was generated by MosSCI insertion into ttTi5606 on chromosome II, and the silenced expression in the intestine was rescued by *met-2* mutation. Data were collected over three independent experiments. Yellow arrowheads indicate intestinal cell nuclei. Scale bar, 50 μm. (b) the percentage of mCherry de-silenced worms in different mutants. A minimum of 20 worms were observed each strain. All the worms observed are at the L4 stage.

To investigate whether intestine-specific silencing is mediated by the RNAi pathway, we examined the expression patterns of reporters in mutants with distinct functions in the RNAi pathway (Figure 2B). The initial step of exo-RNAi involves the processing of dsRNA by the RNase III-related enzyme Dicer and its associated proteins [43–47]. In *C. elegans*, the resulting siRNAs are bound by the Argonaute protein RDE-1, which is necessary for targeted mRNA degradation [48,49]. However, when *rde-1* or *rde-4* is mutated, we observed that the silencing of the *isw-1* and *lin-13* reporters in the intestine remained intact, indicating that the silencing of single-copy transgenes is not caused by a defect in the initial step of exo-RNAi. We then focused on factors involved in the endo-RNAi pathway, and found that those affecting 22 G RNA accumulation play crucial roles in intestine silencing. *mut-16* and other mutator class genes are responsible for secondary endo-siRNA biogenesis [50,51]. Loss of MUT-16 leads to a significant reduction in ERGO-1 class 26 G RNAs and their dependent 22 G RNAs. MUT-16 recruits MUT-2, MUT-15, MUT-14, and MUT-8, with MUT-8 further recruiting MUT-7, to assemble the mutator focus [52]. Deep sequencing of *mut-2*, *mut-7*, and *mut-16* mutants reveals general defects in the accumulation of 22 G RNAs [26,50], Interestingly, the expression levels of the *isw-1* and *lin-13* reporters in the intestine dramatically increase when *mut-2*, *mut-16* or *mut-7* is mutated (Figure 2). Furthermore, consistent with the expression profile of 26 G RNA during development [53], the restoration of expression is most significant at the L4 stage, with minimal observed rescue at the L1 stage (Figure S2). This sequential rescue pattern suggests that the silencing in the intestine is mediated by 22 G RNAs triggered by 26 G RNAs.

### 3.3. Crucial components of the endo-RNAi pathway exhibit high expression levels in the intestine

The *mut-2* gene plays a crucial role in both the endo-RNAi pathway and transposon mobilization [48,54]. *mut-2* encodes a conserved protein in the polymerase β nucleotidyltransferase superfamily [55], which is required for siRNA accumulation and efficient RNAi in all tissues. However, the precise expression pattern of *mut-2* has remained unknown. To investigate this, we generated a single-copy fluorescent reporter line driven by the *mut-2* promoter using the MosSCI method. In the reporter worms, *mut-2* expression was undetectable at the L1 stage but gradually increased from the L2 to the L4 stages in the intestine. The expression levels in other tissues did not show significant change. This sequential expression pattern of *mut-2* is consistent with the observed rescue of reporter expression in the intestine, which is most prominent at L4 stage rather than the L1 stage. Similarly, reporter lines for *mut-7* and *mut-16* were also generated using the same approach. In line with transgene de-silencing observed in the mutant worms, the expression of *mut-7* and *mut-16* reporters was minimal during early developmental stages and increased dramatically at the L3 and L4 stages.

### 3.4. Intestinal silencing of transgenes is impacted by histone modifications

Epigenetic regulation, including chromatin remodelling and post-transcriptional modification, is a conserved mechanism for repressing repetitive elements in most of metazoans [56]. However, it remains unclear whether the same epigenetic regulation affect the single-copy transgenes integrated into the chromosome. In *C. elegans*, MET-2 is one of the histone methyltransferases (HMTs) responsible for mono- and di-methylation of H3 lysine 9 methylation (H3K9me1/2), while SET-25 catalyses the trimethylation of H3K9 (H3K9me3) [57]. Both H3K9me3 and H3K27me3 are associated with silent chromatin domains [58–60]. Consistent with prior studies, we observed an increase in the expression of the single-copy transgene of *lin-13* in *met-2* mutants.

**Figure 3.**
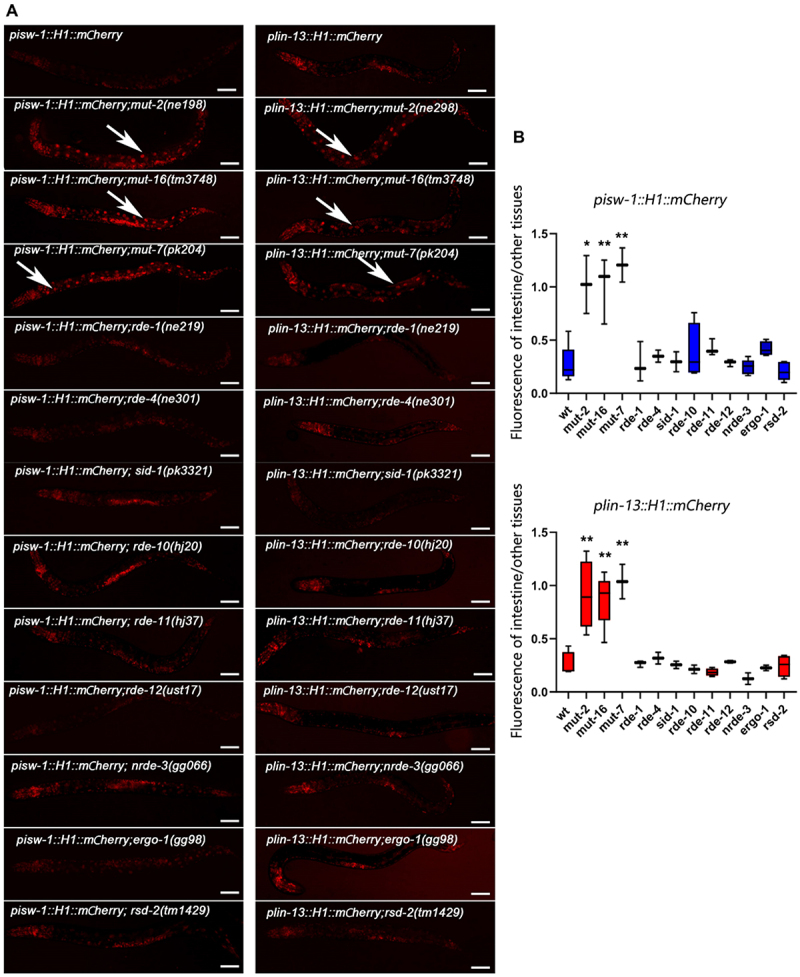
MosSCI transgenic *lin-13* and *isw-1* expression levels were regulated by the RNAi pathway. (a) Live worm images exhibited a differentially expressed level of reporters in the intestines of different mutant worms. (b) Relative fluorescent signals of the intestine compared to other tissues. Data were collected over three independent experiments. All worms observed are at the L4 stage. The anterior is on the left of the images. Data were collected over three independent experiments. White arrows indicate the intestine nuclei. Scale bar, 50 μm. Unpaired *t*-tests performed for comparisons; *, *p* < 0.1. **; *p* < 0.01.

In contrast to the observed effect of *met-2* on intestine silencing (Figure 3), we found that *met-1*, which encodes an H3K36 methyltransferase [61], exhibits no influence on the silencing in the intestine (Figure 3). Additionally, *hda-3*, encoding a histone deacetylase known to impacts endogenous gene repression [19,62], showed no effect on the intestinal silencing observed in this study.

### 3.5. The balance of primary-siRnas and secondary-siRnas amplification throughout transgene regulation

**Figure 4.**
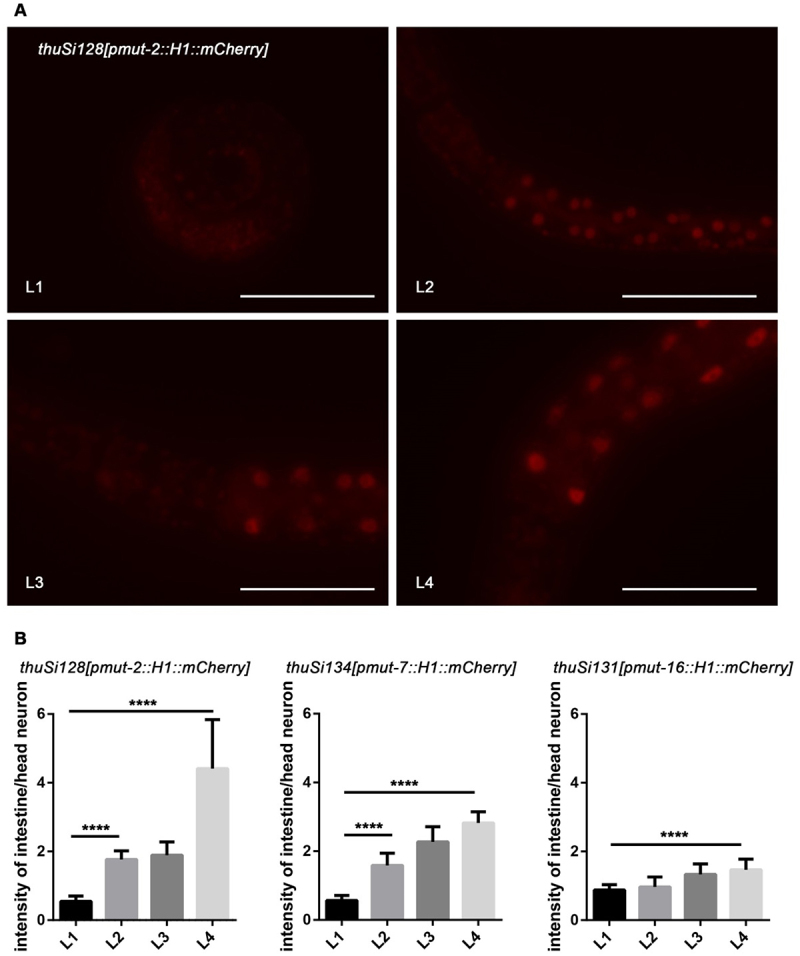
*Mut-2*, *mut-7*, and *mut-16* increased their expression level during the larval development. (a) Images of the anterior and partial intestine of the *mut-2* reporter. From L1 to L4, strengthened intestinal expression of the single-copy transgenic reporter of *mut-2* was detected, while the fluorescence intensity of head neurons was stable. Scale bar, 50 μm. (b) The relative fluorescent signal of the intestine to head neurons increased significantly. Data were collected over three independent experiments. The data are presented as mean + SEM. Unpaired *t*-tests performed for comparison; ****, *p* < 0.0001.

The 26 G RNAs, also known as primary siRNAs, are produced by RNA-directed RNA polymerase (RdRP) and target a significant portion of the *C. elegans* genome. Following the biogenesis of primary siRNAs, 22 G RNAs are triggered known as secondary siRNAs. These 22 G RNAs act as effectors in regulating the expression of the target genes. Studies have shown that the levels of 26 G RNAs changes as sequential rounds [53]. Interestingly, although the expression level of 26 G RNAs decreases from the L1 to L4 stage, the transgene silencing in the intestine remains unchanged during different stages of *C. elegans* development (Figure S2). However, at later stages of development, the expression level of *mut* genes, responsible for the amplification of secondary siRNAs, increases(Figure 4). This suggests that these two components of the endo-siRNA pathway cooperate to regulate transgene silencing in the intestine. To test whether primary siRNAs play a fundamental role in transgene silencing during the early stages of *C. elegans* intestine, we examined the expression of reporters in RdRP mutants. The RRF-1 complex is responsible for RdRP activity in *C. elegans*. Interestingly, we found that *rrf-1*, which is directly involved in the biogenesis of secondary siRNAs, does not affect the intestinal silencing of *lin-13* or *isw-1*. This finding is consistent with previous reports that EGO-1 can compensate for RRF-1 in the production of *mut*-dependent germline 22 G RNAs [26].

## 4. Discussion

In this study, we have demonstrated tissue-specific silencing of single-copy transgenes in *C. elegans*. Unlike the silencing of artificially created repetitive DNA [2], which may be attributed to the recognition of arrays as repetitive or heterochromatin-like structures [41], the silencing of single-copy transgenes is regulated by RNAi pathway. Previous studies have shown a correlation between the exo-RNAi pathway and transgene silencing in the intestines of *C. elegans*, but those studies utilized multi-copy transgenic strains as reporters [10]. Our findings provide evidence for specific regulation of transgene silencing in a tissue-specific manner.

The majority of knowledge regarding the RNAi pathway in *C. elegans* stems from studies conducted in the germline, with the understanding extrapolated to somatic tissue. When RNAi is performed through feeding or soaking, the intestine of *C. elegans* is considered the primary site for the uptake and transport of double-stranded RNA from the environment. The transporter extensively studied in this context is SID-1, which is responsible for the systemic transportation of dsRNA. However, in our study, we observed no restoration of fluorescence in reporter lines when using *sid-1* mutant worms. Although the intestine plays a crucial role in exo-RNAi function, it remains unclear whether genes expressed in the intestine are regulated by endo-RNAi or not.

The RNAi pathway is not essential for the survival of *C. elegans*, as many of the factors involved in this RNAi pathway are not required for normal functioning of organism. This characteristic makes *C. elegans* an ideal model organism for investigating the mechanisms of RNAi. The endo-RNAi pathway, which regulates the expression of numerous genes in *C. elegans*, plays a vital role in maintaining cellular the homoeostasis.

MosSCI-mediated single-copy insertion transgenes, consisting of germline-expressed genes, can be silenced through piRNA-mediated mechanisms, which are predominantly expressed in the germline [63,64]. However, the regulation of single-copy transgenes in somatic cells remains unclear. In this study, we provide evidence that endo-siRNAs might regulate the expression of the single-copy transgenes in somatic tissues. Two distinct populations of small RNAs are proposed to participate in RNAi: primary siRNAs and secondary siRNAs. Previous investigations have shown that secondary siRNAs make up the majority and likely play a crucial role in destabilizing targeted transcripts [23,65]. The biogenesis of primary siRNAs and the amplification of secondary siRNAs likely work together to finely regulate target genes. During initial siRNA generation, the small RNAs are short and lack significant specificity, resulting in the targeting of multiple genes. However, the production of secondary siRNAs, which is crucial for the degradation of accumulated mRNA, involves a complex composed of multiple proteins. These proteins involved in secondary siRNA amplification exhibit distinct spatiotemporal expression patterns, allowing for precise regulation of target genes.

Unlike dsRNA-triggered gene silencing, which leads to the degradation of target mRNA through siRNA and associated proteins, endogenous siRNAs can also induce gene silencing at transcriptional level in *C. elegans*. Endo-siRNAs impact histone modifications (such as H3K9me3 and H3K7me3) [66–68], resulting in transcriptional repression at specific target loci [69]. HRDE-1 associates with H3K9me3 at RNAi-targeted loci and maintains germline silencing [70]. In *Arabidopsis*, the silencing of repetitive transgenes decreases in various backgrounds, including the DDM1 mutation, which is thought to encode a SWI2/SNF2-like protein involved in chromatin remodelling [71–73]. In *C. elegans*, several gene mutations have been identified as necessary for silence repetitive transgenes, such as *mes-2*, *mes-3*, *mes-4*, *mut-7*, *rde-2*, and others [11,54,74], some of which are related to chromatin structure. Conversely, *pals-22* is required to prevent the silencing of repetitive transgenes in neurons and other somatic tissues, and this process depends on small RNA biogenesis of small RNAs [75]. In our study, the *met-2* mutation rescued the intestinal silencing of the *lin-13* reporter, but it had no effect on the *isw-1* reporter. This suggests that, histone modifications may not be sufficient to alter the landscape of intestinal silencing. The genes *mut-2*, *mut-7*, and *mut-16* are components of the mutators pathway, which is involved in the amplification of endo-siRNAs [50]. The involvement of mutator genes in intestinal silencing suggests that the silencing of single-copy transgenes shares mechanisms with the silencing of repetitive DNA to some extent.

The *mut-7* gene, encodes a protein with homology to RNaseD, and was initially identified for its ability to activate transposons in the germline of *C. elegans*. *mut-7* mutants have been shown to be resistant to RNAi [54], and the transposition of *mut-2(r459)* mutants was further enhanced [76]. These mutants, where transposons (such as Tc1) are activated in the germline, are referred to as mutators. Tabara et al. demonstrated that the activation of transposons by mutator genes is genetically linked to the RNAi pathway [48]. Previous studies have revealed that MUT-7 acts downstream of siRNA production [52]. MUT-16 is required for multiple endogenous germline and somatic siRNA pathways [50]. Our study is consistent with previous findings regarding the role of mutators in RNAi function, and we extend this effect to somatic tissues. Additionally, in *mut-2*, *mut-7*, and *mut-16* mutant worms, the expression of single-copy transgenes was detected in the germline but not in the wild-type worms (data not shown), indicating shared characteristics between the intestine and the germline.

Given the wide range of silencing effects observed across different organisms, it is crucial to unravel the mechanisms underlying transgene silencing in the intestine. In *C. elegans*, the intestine serves as a metabolic organ, playing roles analogous to the liver and adipose tissue in other organisms. It also participates in communication with the germline through lipophilic-hormone signalling, which can impact the ageing process [77]. The intestinal cells of *C. elegans* may serve as a component of the defence system, and similar functions have been studied in vertebrates [78]. For instance, immune cells producing IgA have been found to travel from the gut to the brain in mice, suppressing neuroinflammation [79].

Artificially designed repeated DNA sequences (reporters) are commonly used to visualize the expression patterns of specific genes, especially when the expression signal is negligible or undetectable using single-copy transgenes or endogenous genes. However, our study reveals that single-copy transgenes of certain genes may exhibit similar expression patterns to their endogenous counterparts, but they do not faithfully reflect the natural regulation of gene expression within the organism. The native context of a gene is crucial for the precise regulation of its expression, and chromosomal modifications might play a vital role in this process.

In conclusion, our study demonstrated that endo-siRNA can facilitate the transgenic silencing of the intestine at early stage of worm development by regulating the expression of *mut-2*, *mut-7* and *mut-16*. At present, lack of functional data remains a major limitation of this study. Therefore, we attempt to validate the function of intestine when endo-siRNA pathway was defected.

## Supplementary Material

Supplemental Material

## Data Availability

The authors confirm that the data supporting the findings of this study are available within the article and its supplementary materials.
