# Supplementary figures and images for: Tissue-specific silencing of integrated transgenes achieved through endogenous RNA interference in Caenorhabditis elegans
Source: RNA Biol. 2024 Mar 26;21(1):1–10. doi: 10.1080/15476286.2024.2332856 (PMC10978027; doi:10.1080/15476286.2024.2332856)

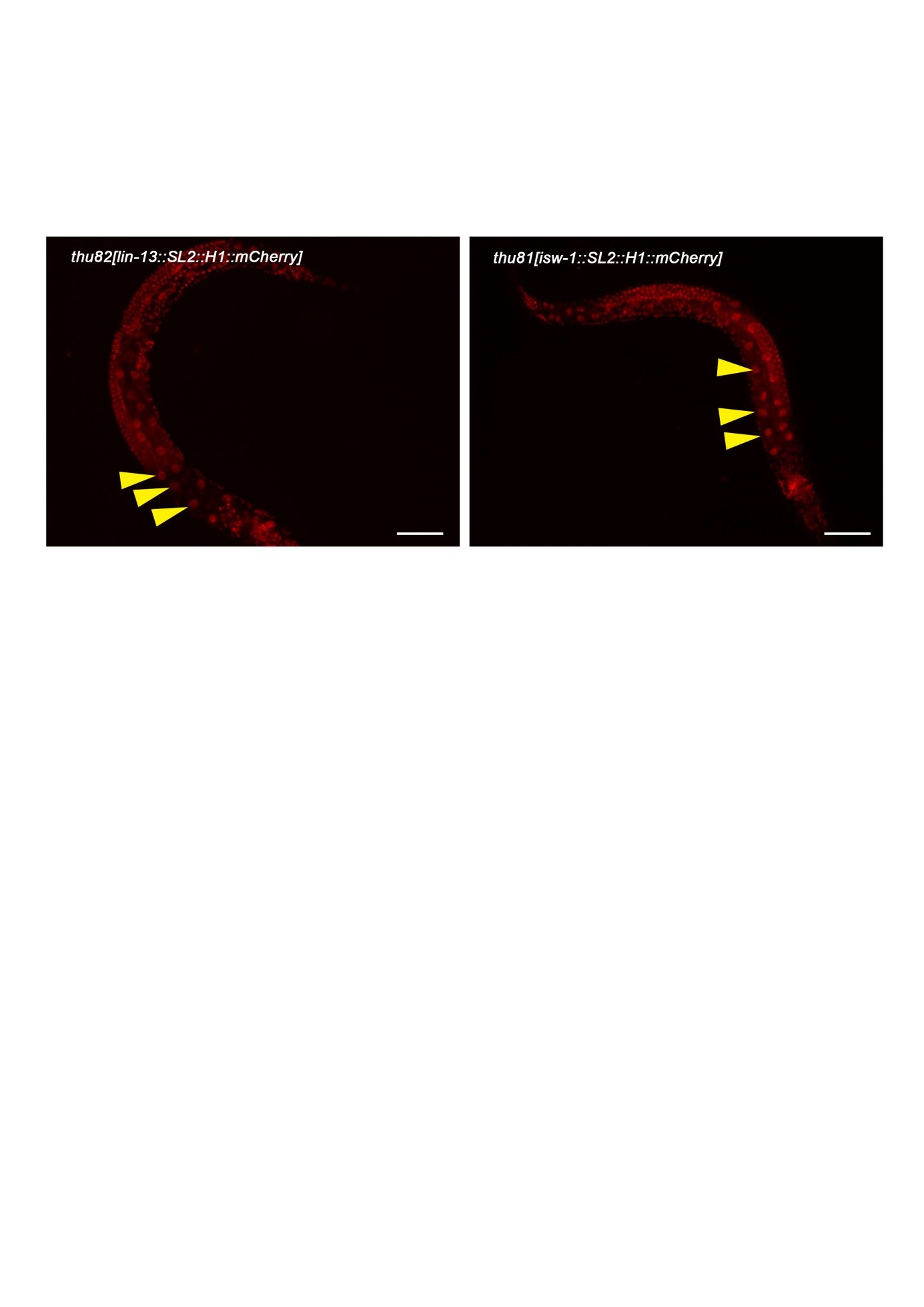

Supplement: Supplemental Material [file KRNB_A_2332856_SM5859.zip › figure S1.jpg]

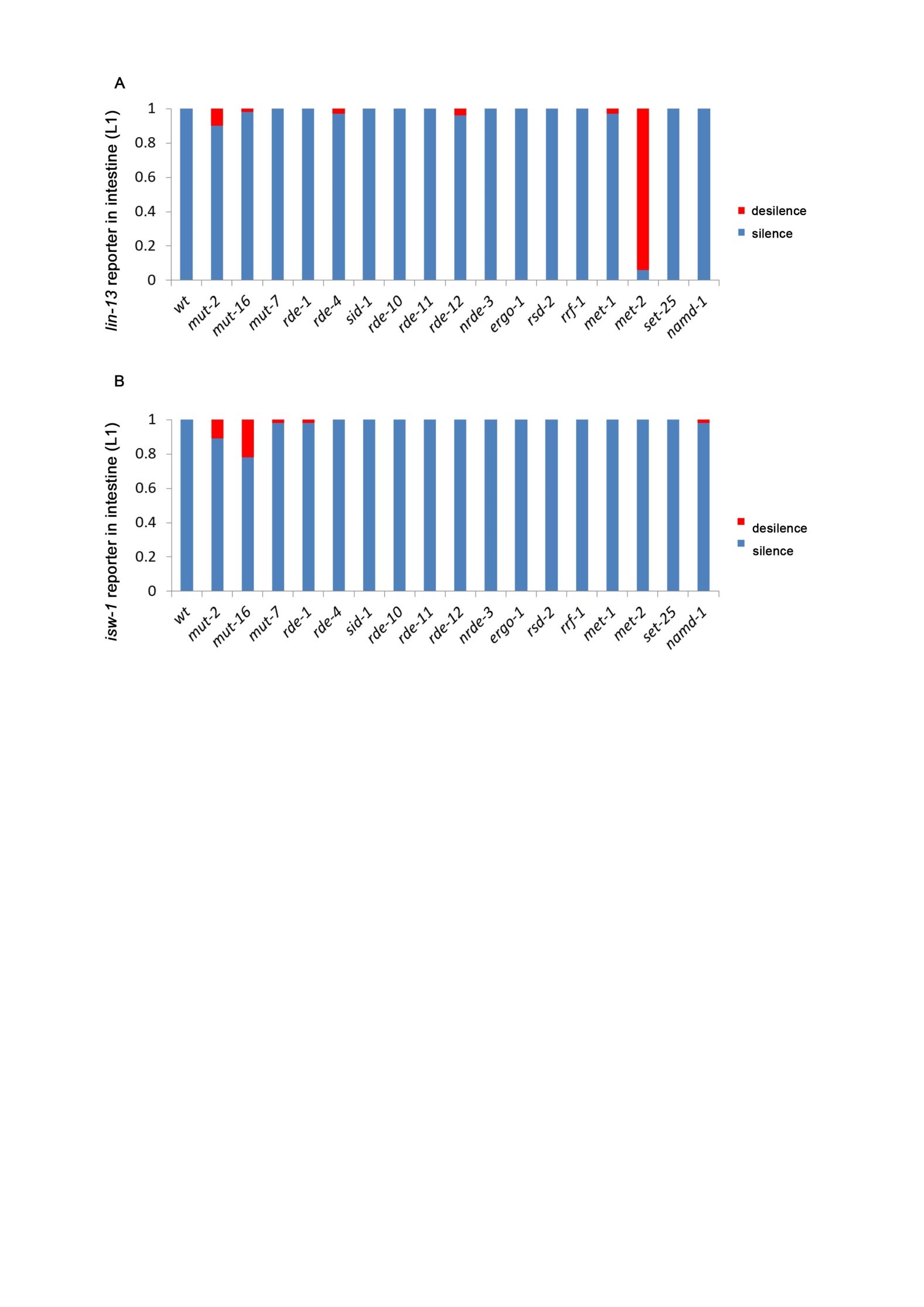

Supplement: Supplemental Material [file KRNB_A_2332856_SM5859.zip › figure S2.jpg]
